# Epinephrine, inodilator, or no inotrope in venoarterial extracorporeal membrane oxygenation implantation: a single-center experience—an RCT would be desirable

**DOI:** 10.1186/s13054-019-2712-2

**Published:** 2020-01-22

**Authors:** Romain Jouffroy, Benoit Vivien

**Affiliations:** SAMU de Paris, Service d’Anesthésie Réanimation, Hôpital Universitaire Necker - Enfants Malades, Assistance Publique - Hôpitaux de Paris, and Université Paris Descartes - Paris 5, Paris, France

To the Editor:

In the 2019 September issue of the *Journal*, Zotzmann et al. [1] reported an association between epinephrine therapy within the first 24 h after cannulation for VA-ECMO and poor survival compared to patients with or without any inodilator therapy. Firstly, we compliment the authors for this very interesting study. Nevertheless, in our opinion, some methodological issues deserve their attention.

We are surprised that 41% analyzed patients received no inotropic agents while as stated by the authors these agents aim to secure left ventricular ejection in order to decrease the risk of intra-cardiac stasis [2] in patients benefiting from VA-ECMO for cardiogenic shock or cardiac arrest. The norepinephrine dose requirement reflects the severity of the underlying hemodynamic failure despite VA-ECMO, independently of the associated inotropic agent [3]. We cannot exclude a selection bias; patients with a higher risk of unfavorable evolution are those requiring higher doses of catecholamines including epinephrine. Furthermore, factors were known at the time of treatment instauration and might have influenced the physician’s choice of the inotropic agents.

Beyond this, essential factors directly influencing the outcome, i.e., low and no-flow duration [4], are not included in the analyses and may directly impact the results. As stated by the authors, the number of VA-ECMO for cardiac arrest was higher in the epinephrine group.

Finally, we believe that the choice of catecholamines is related to the indication, cardiogenic shock or cardiac arrest, of VA-ECMO support and needs randomized control trials [5].

## Authors’ response

Viviane Zotzmann, Daniel Duerschmied and Dawid L. Staudacher

We thank Drs. Jouffroy and Vivien for their interest in our article [1]. We have to agree to the constructive criticism highlighting a potential bias in our retrospective data. Our key finding that continuous epinephrine administration in patients requiring venoarterial extracorporeal membrane oxygenation (VA-ECMO) support was associated with adverse outcome should therefore be considered hypothesis-generating [1].

We also agree that there are in vitro data on intra-cardiac stasis in LVAD-assisted artificial hearts [2]. This does however not necessarily mean that outcome in patients on VA-ECMO can be improved by inotropic therapy, and therefore has to be evaluated. Same holds true for generalizing retrospective data on norepinephrine dose derived from pediatric and neonatal patients [3] to adults, or for data from patients after out-of-hospital cardiac arrest [4] to a collective of mixed in-hospital and out-of-hospital arrest patients after extracorporeal resuscitation (eCPR). While randomized trials on VA-ECMO in patients with cardiogenic shock [5] are in dire need, they will not answer the pressing questions concerning optimal treatment of patients put on VA-ECMO. To the best of our knowledge, no other registry or registered prospective trial evaluates the use of epinephrine in patients requiring VA-ECMO support.

The presented data [1] are therefore timely and warrant further discussion. We are grateful for Drs. Jouffroy and Vivien’s enthusiasm and hope for future collaboration. We fully support and urge for randomized trials in VA-ECMO patients to study treatment strategies.

## Data Availability

Not applicable.
